# Pre-apoptotic response to therapeutic DNA damage involves protein modulation of Mcl-1, Hdm2 and Flt3 in acute myeloid leukemia cells

**DOI:** 10.1186/1476-4598-6-33

**Published:** 2007-05-11

**Authors:** Line Wergeland, Gry Sjøholt, Ingvild Haaland, Randi Hovland, Øystein Bruserud, Bjørn Tore Gjertsen

**Affiliations:** 1 Institute of Medicine, Hematology Section, University of Bergen, N-5021 Bergen, Norway; 2 Department of Medicine, Hematology Section, Haukeland University Hospital, Bergen, Norway; 3 Center for Medical Genetics and Molecular Medicine, Haukeland University Hospital, Bergen, Norway; 4 Proteomic Unit, Department of Biomedicine, University of Bergen, Bergen, Norway

## Abstract

**Background:**

Acute myeloid leukemia (AML) cells are characterized by non-mutated *TP53*, high levels of Hdm2, and frequent mutation of the Flt3 receptor tyrosine kinase. The juxtamembrane mutation of *FLT3 *is the strongest independent marker for disease relapse and is associated with elevated Bcl-2 protein and p53 hyper-phosphorylation in AML. DNA damage forms the basic mechanism of cancer cell eradication in current therapy of AML.

Hdm2 and pro-apoptotic Bcl-2 members are among the most intensely induced genes immediately after chemotherapy and Hdm2 is proposed a role in receptor tyrosine kinase regulation. Thus we examined the DNA damage related modulation of these proteins in relation to *FLT3 *mutational status and induction of apoptosis.

**Results:**

Within one hour after exposure to ionizing radiation (IR), the AML cells (NB4, MV4-11, HL-60, primary AML cells) showed an increase in Flt3 protein independent of mRNA levels, while the Hdm2 protein decreased. The *FLT3 *mutant MV4-11 cells were resistant to IR accompanied by presence of both Mcl-1 and Hdm2 protein three hours after IR. In contrast, the *FLT3 *wild type NB4 cells responded to IR with apoptosis and pre-apoptotic Mcl-1 down regulation. Daunorubicin (DNR) induced continuing down regulation of Hdm2 and Mcl-1 in both cell lines followed by apoptosis.

**Conclusion:**

Both IR and DNR treatment resulted in concerted protein modulations of Mcl-1, Hdm2 and Flt3. Cell death induction was associated with persistent attenuation of Mcl-1 and Hdm2. These observations suggest that defining the pathway(s) modulating Flt3, Hdm2 and Mcl-1 may propose new strategies to optimize therapy for the relapse prone *FLT3 *mutated AML patients.

## Background

Anthracyclines like daunorubicin (DNR) are used in the induction treatment of acute myeloid leukemia (AML), obtaining short time complete hematological remission for more than 65% of adult AML patients with *de novo *AML [1]. Successful hematological remission after only one induction cycle is a favorable prognostic parameter and is associated with decreased risk of later AML relapse [1,2]. Induction therapy causes rapid activation of the tumor suppressor p53 followed by dominating p53-targeted gene expression *in vivo *[3]. A major mechanism for this p53 induction is DNA damage through anthracycline-stabilization of the DNA:topoisomerase II complex [4], but cell death induction by anthracyclines may also involve other molecular targets independent of p53 [4-7].

Ionizing radiation (IR) is frequently used in the treatment of solid cancers, in the conditional treatment before allotransplantation of leukemia patients and in radioisotope-conjugated therapeutic antibodies directed against AML cells [8,9]. IR and anthracyclines induce growth arrest and cell death through DNA-damage, but also involve cell membrane-related effects in regulation of apoptosis [4-7,10]. We have previously reported that AML patient cells respond with varying sensitivity to IR-induced proliferation arrest [11], and it may therefore be of interest to determine molecular mechanisms for radioresistance in more detail.

The strongest molecular predictor for AML relapse is internal tandem duplications in the juxtamembrane domain of the receptor tyrosine kinase Flt3 (Flt3-ITD). These mutations are present in approximately one third of the patients [12]. Flt3-ITD are associated with increased DNA repair [13], an observation suggesting that these cells are able to recover from DNA damage caused by topoisomerase II blockage and thus have a more drug-resistant phenotype. The expression of anti-apoptotic Bcl-2 protein family members is also influenced by the mutational status of Flt3 [14]. We have recently shown that a duplication of Y591 in Flt3-ITDs is associated with elevated Bcl-2 protein and hyper-phosphorylated wild type (wt) p53 in AML, proposing a mechanism for inactivation of p53 [14].

Mcl-1 is an anti-apoptotic member of the Bcl-2 family of proteins. High levels of Mcl-1 have been detected in cells from patients with relapsed AML [15]. Therapeutic targeting of Bcl-2 family proteins seems to depend on Mcl-1 to trigger apoptosis [16]. It may therefore be of particular interest to examine the Mcl-1 modulation in DNA damage therapy.

In contrast to solid tumors, more than 90% of the AML cases comprise wild type p53 [17,18]. On the other hand, the E3 ubiquitin ligase Hdm2 is usually strongly expressed in AML, contributing to block the growth inhibitory and pro-apoptotic effect of p53 [19]. IR induces DNA damage and rapid down regulation of Hdm2 through induction of auto-ubiquitination and subsequent proteasomal degradation [20]. Recent reports indicate that Hdm2 have important p53-independent activities, including regulation of cell membrane receptors like insulin-like growth factor (IGF) 1 receptor and β2-adrenergic receptor through ubiquitination [21]. However, it is not known whether the Flt3 receptor is regulated by Hdm2.

Concerted protein modulation of a receptor tyrosine kinase, the E3 ubiquitin ligase Hdm2 and selected Bcl-2 family members through DNA damage therapy has previously not been reported. Our study indicated that both IR and DNR induced Hdm2 protein down regulation, partly Flt3 protein elevation, and a pro-apoptotic shift in the expression of proteins in the Bcl-2 family. Flt3 and Hdm2 might have a reciprocal regulation at the protein level and *FLT3 *mutations could be involved in protection against IR-induced apoptosis through a persisting Mcl-1 level.

## Results

### Ionizing radiation induces reciprocal regulation of Flt3 and Hdm2 protein in NB4 cells

**Figure 1:**
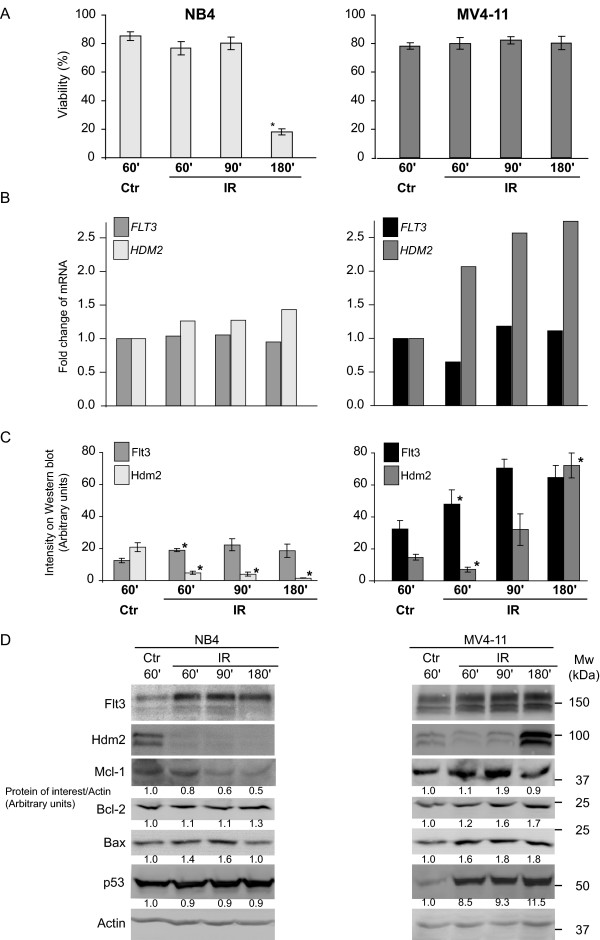
**Rapid IR-induced protein modulation of Flt3, Hdm2 and Bcl-2 family members in AML cell lines**. **A**. Cells were exposed to 25 Gy and fixed after the indicated time (minutes). The percentage of normal nuclei in a total of 200 cells was determined in Hoechst stained cells for each time point. The results shown represent the mean of three separate experiments and the error bars show standard error of mean (SEM = Standard Deviation/√n). The star denotes statistical significance relative to the control, this is determined by a Students two-tailed *t*-test, p < 0.05. **B**. mRNA level of *FLT3 *and *HDM2 *was determined by Real-time PCR in one typical experiment. GAPDH was used as endogenous control. **C**. IR down regulated Hdm2 protein and up regulated Flt3 protein in these AML cell lines. The diagram shows measured intensity on three separate Western blots (normalized to Actin). The error bars show standard error of mean SEM and the stars represents significance as in **A**. **D**. Visualization of the protein modulations of Flt2 and Hdm2 shown in **C**. in addition to modulation of proteins in the Bcl-2 family. Mean intensity on the Western blots written below the corresponding panel were measured and normalized to Actin and to the control. The values shown are arbitrary units and represent one typical experiment.

The promyelocytic cell line NB4 is characterized by mutated *TP53 *and non-functional p53 protein [22,23] as well as wild type *FLT3 *[24]. DNA damaging 25 Gy IR of NB4 cells resulted in increased apoptosis, but no modulation of *FLT3 *or *HDM2 *mRNA was observed (Fig. 1a,b; left panel). Hdm2 responds to IR with protein auto-degradation [20], and it regulates endocytosis of certain receptors like the IGF 1 receptor [25]. We examined Flt3 and Hdm2 at different time points after IR (25 Gy) and found highly significant reciprocal regulation at the protein level (Fig. 1c; left panel). This was accompanied by attenuation of the anti-apoptotic Mcl-1, an increase in Bax but unaltered Bcl-2 (Fig. 1d; left panel) and Bcl-X_L _(data not shown). Previous studies have shown that DNA-damaging *in vivo *chemotherapy of AML has no effect on *MCL-1 *gene induction, but rapidly induces *BAX *and *PUMA *mRNA [3] (Øyan et al., manuscript in preparation). p21 protein was not detected in NB4 cells (data not shown), and the p53 protein level was not altered after irradiation (Fig. 1d; left panel), reflecting its non-functional status.

### Hdm2 response and stable Mcl-1 in the IR-resistant cell line MV4-11

MV4-11 is characterized by *FLT3-ITD*, loss of wilt type *FLT3 *allele, and wild type *TP53 *[22,24]. MV4-11 cells were resistant to IR with regards to apoptosis induction (Fig 1a, right panel), but responded with more than one fold increase in *HDM2 *mRNA (Fig. 1b), reflecting the functional p53. The level of Hdm2 protein showed a small but significant decrease after 60 minutes before an increase was detected, whereas the Flt3 level increased in response to IR and was not attenuated by the elevated *HDM2 *level after 180 minutes (Fig. 1c,d). Another striking difference from the NB4 cells with *FLT3-wt *was that the Mcl-1 level did not change in response to IR (Fig. 1d). Furthermore, MV4-11 responded to IR with increased protein levels of p53, Bax, Bcl-2, (Fig. 1d) and p21 (data not shown) while the level of Bcl-X_L _was unaltered (data not shown). The IR induction of p53, Hdm2, Bax and p21 suggests that the p53 transcriptional activation in MV4-11 is intact [3].

### Attenuation of Hdm2 and Mcl-1 is independent of p53 and Flt3

**Figure 2:**
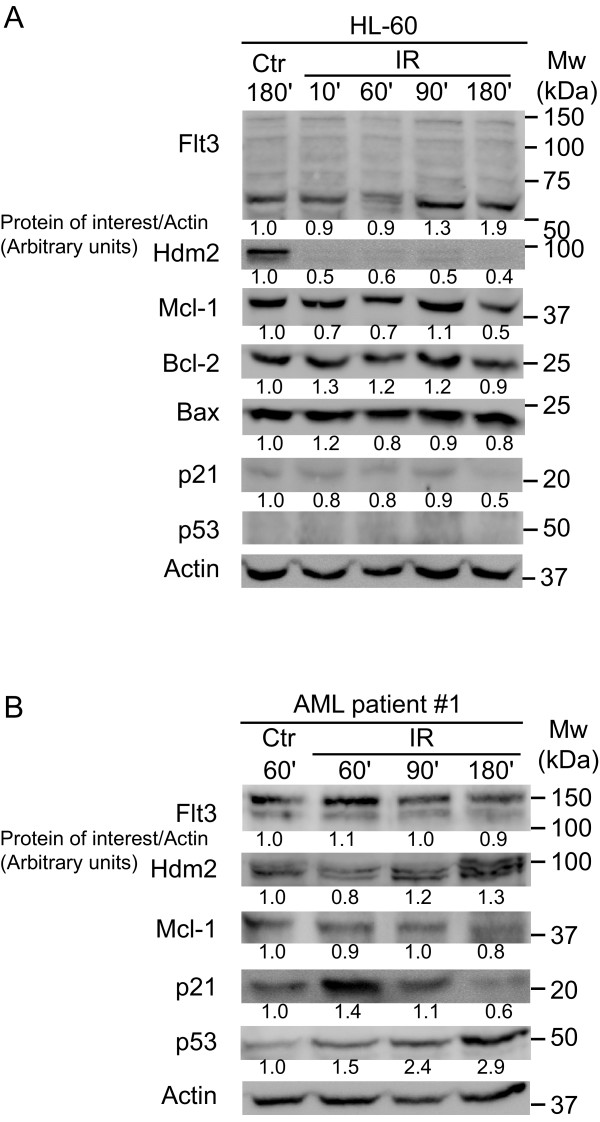
**IR decrease Hdm2 protein in HL-60 (p53 -/-) and in primary AML cells**. **A**. HL-60 cells treated with 25 Gy and harvested at indicated time (minutes) demonstrated a rapid decrease in Hdm2 protein after IR. There was no detectable Flt3 protein at 130 kDa. **B**. Primary AML cells also treated with IR and harvested at indicated time, demonstrated the rapid Flt3 increase and Hdm2 attenuation as previously observed. This was followed by an increase in Hdm2, reflecting functional and elevated p53 protein. Mean intensity on the Western blots were measured and normalized to Actin and to the control. Values shown are arbitrary units and represent one typical experiment.

The effect of IR was also examined in HL-60 cells, characterized by wild type *FLT3 *and deleted alleles for *TP53 *[22,24]. Like inNB4 and MV4-11 cells, Hdm2 was attenuated and Flt3 increased, but the Flt3 protein appeared not to be full length (Fig. 2a; ~150 versus ~60 kDa). The lack of full length Flt3 was confirmed in cells from both ATCC and DSMZ within four passages of culture, and immunoprecipitation of Flt3 in these cells did not reveal any low molecular anti-Flt3 reactive form (data not shown). Flt3 protein has previously been reported non-detectable in HL-60 cells [26].

Bcl-2 family members showed no significant response to IR in HL-60 cells except a late decrease in Mcl-1.

Human primary AML cells (patient 1) were irradiated and examined for Flt3 and Hdm2 modulation (Fig. 2b), indicating that the reciprocal Flt3-Hdm2 response to DNA damage also could be present in primary leukemia cells. In contrast to the HL-60 cells where the p21 response was absent, early increase was present in the primary AML cells. These differences reflects an absence of a p53 response in HL-60 cells and a presence of such in the patient cells (Fig. 2a,b).

### Daunorubicin induces attenuation of Hdm2 and Mcl-1 independent of *TP53 *and *FLT3 *status

**Figure 3:**
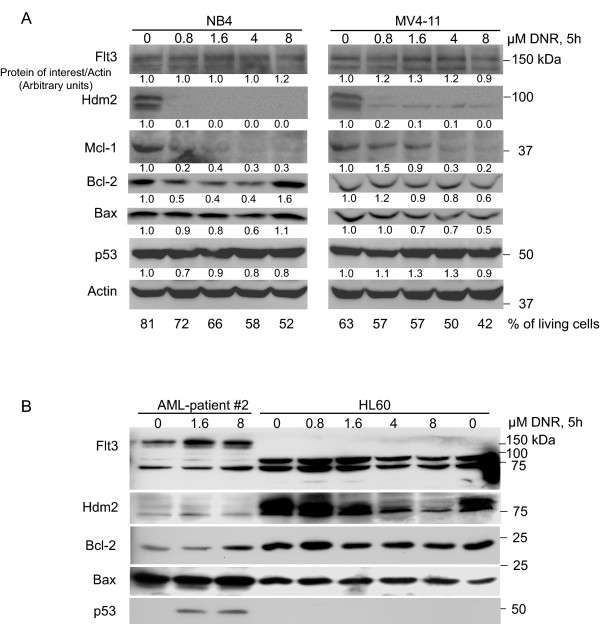
**Daunorubicin therapy of primary AML cells and cell lines increased Flt3 protein and attenuated Hdm2/Mcl-1**. **A**. Treatment of NB4 and MV4-11 cells with DNR resulted in an increase in Flt3 and a decrease in Hdm2. The mean intensity on one representative Western blot was calculated and normalized to Actin and to the control. The numbers shown are in arbitrary units and represent one typical experiment. The percentage of living cells was determined by flow cytometry. The living cells distinct forward and side scatter properties were used to separate viable cells from dead cells. **B**. Increasing doses of DNR induce Flt3 and down regulate Hdm2 protein in primary AML cells *in vitro*. Note that Hdm2 is down regulated in HL-60 cells, a cell line with lack of full length Flt3 protein and with deleted alleles for p53. All wells on the SDS-PAGE gel was loaded with equal amounts of protein, and Coomassie staining of the gel after blotting confirmed this equal loading.

Since both DNR and IR induce DNA damage, we examined the effect of DNR in both AML cell lines (NB4, MV4-11 and HL-60) and primary cells (patient 2). The cells were treated *in vitro *(Fig. 3) with DNR for 5 hours at relevant concentrations [3]. The NB4 and MV4-11 cell lines were sensitive to DNR with regards to apoptosis induction, and both Mcl-1 and Hdm2 were down regulated (Fig. 3a). Although DNR increased Flt3 protein in all the AML cells tested (Fig. 3ab), this effect was most prominent in MV4-11 cells, HL-60 and the primary AML cells. HL-60 cells showed an increase in putative short forms of Flt3 protein with low doses of DNR (Fig. 3b).

### Flt3 and Hdm2 protein are reciprocally regulated *in vivo*

**Figure 4:**
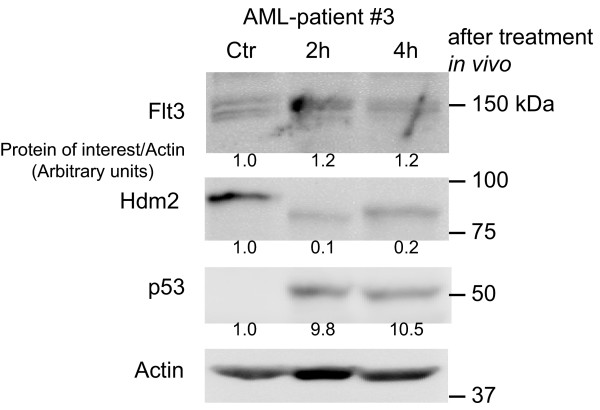
**Induction therapy of AML reciprocally regulates Flt3 and Hdm2 proteins *in vivo***. AML cells sampled from a patient undergoing induction chemotherapy with an anthracycline and cytarabin were subjected to Western blotting and analyzed for Flt3, Hdm2 and p53 expression. The mean intensity on one representative Western blot was calculated and normalized to Actin. The numbers shown are in arbitrary units.

We have recently demonstrated swift induction of p53 and Bax proteins in AML cells collected from patients undergoing induction chemotherapy with anthracyclines and cytarabine [3]. It was therefore of interest to examine the AML cells' protein levels of Flt3 and Hdm2 after *in vivo *chemotherapy (Fig. 4, one representative patient). AML cells from patients were collected within the first 4 hours of chemotherapy, and showed a strong *in vivo *decrease in Hdm2, in addition to increase in p53 and Flt3.

## Discussion

**Figure 5:**
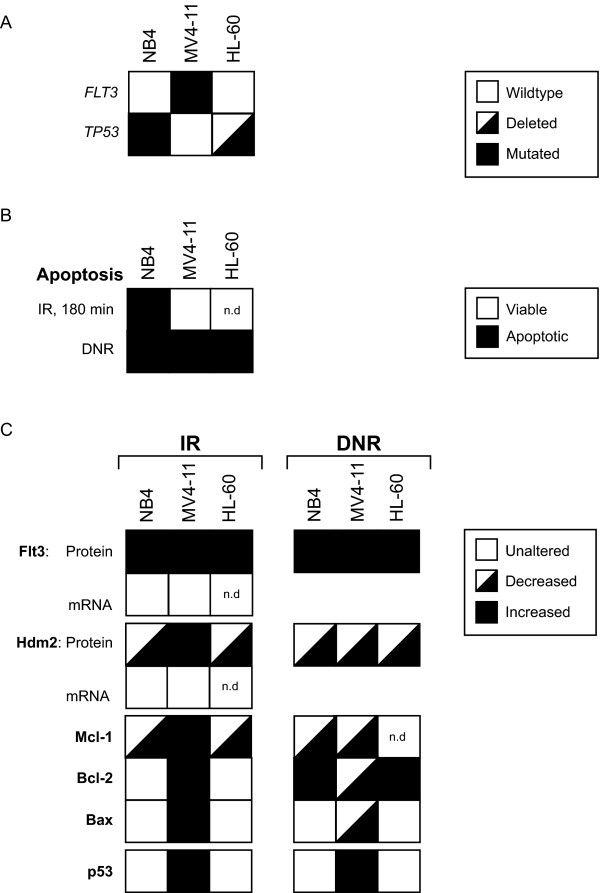
**Summary of the results**. **A**. *FLT3 *and *TP53 *mutational status of the cell lines used in this study. **B**. A summary of the ability of IR and DNR to induce apoptosis in the three different cell lines studied (n.d; not determined). **C**. Overview of the concerted protein modulations elicited by DNA-damaging therapy found in this study (n.d; not determined).

We demonstrated that Flt3 protein increased in response to IR and DNR in all AML cell lines and primary leukemic cells tested, *in vitro *and *in vivo*. Likewise, Hdm2 was down regulated in concert with the Flt3 increase. This reciprocal regulation was consistent in all experiments except in MV4-11 cells 3 hours after IR and in NB4 cells treated with DNR. A summary of all the results is shown in Fig. 5. Several scenarios may explain this mutual modulation of Flt3 and Hdm2. The IGF 1 receptor, a more distant relative of Flt3, has been demonstrated to undergo Hdm2-dependent ubiquitination and degradation [25]. If Hdm2 regulates the turnover of Flt3 through ubiquitination, IR-induced Hdm2 degradation will result in elevated levels of Flt3. The observed down regulated Hdm2 in irradiated AML cells (Fig. 5) is probably due to proteasomal degradation [27,28]. In addition to its ability of auto-ubiquitination, Hdm2 is ubiquitinated by several E3 ubiquitin ligases, including the p300-CBP associated factor (PCAF) and TSG101 [29,30]. Future work is needed to address if modulation of Flt3 level may affect the level of Hdm2, and if this possible action is directly mediated by Flt3 on Hdm2 or involves other E3 ligases.

It can not be ruled out that the increase in Flt3 protein after IR is based on mechanisms independent of Hdm2. IR has been shown to increase the mRNA and protein levels of epidermal growth factor (EGF) receptor as well as the cell surface protein expression of IGF 1 receptor [31,32]. Such mRNA regulation of Flt3 after IR was not observed in our study (Fig. 5).

The NB4 cells, in contrast to the MV4-11 cells, showed IR induced apoptosis (Fig. 5) and a lack of increase in *HDM2 *mRNA level. Since NB4 cells have non-functional p53 [33], this suggests that NB4 undergoes a p53-independent apoptosis during IR-exposure. A possible explanation for the IR-resistance of MV4-11 is that AML cells with Flt3-ITD can repair double-stranded breaks in DNA more efficient than in cells with wild type Flt3 [13], but an anti-apoptotic effect on p53 by the MLL-fusion products may be an alternative mechanism [34]. This makes MV4-11 more protected against apoptosis induced by IR. Other explanations for early IR-induced apoptosis in NB4 cells in contrast to in the MV4-11 cells could include a pro-apoptotic response on the Bcl-2 family members and a lack of Hdm2 induction (Fig. 5). No shift in the balance of Bcl-2/Bax was observed (Fig. 5), thus our data suggest that Mcl-1 is a central player in regulation of DNA-damage induced cell death. A striking feature of IR treated NB4 cells, as well as DNR treated NB4 and MV4-11 cells, was the Mcl-1 down regulation accompanied by apoptosis. These observations emphasize the putative importance of Mcl-1 in regulation of apoptosis in AML, with possible implications for the biology behind disease relapse [15,16].

MV4-11 cells were resistant to IR while DNR effectively induced apoptosis (Fig. 5). DNR elicited a lasting Hdm2 and Mcl-1 down regulation in contrast to IR. This suggests that DNR ignites apoptosis through more pathways than IR and that the Mcl-1 attenuation is a pre-apoptotic event. In addition to the induction of DNA damage, DNR is known to stimulate the level of the second messenger ceramide by *de novo *synthesis and thus trigger apoptosis [5]. Anthracyclines may also induce apoptosis via signalling through altered plasma membrane lipid rafts and the death receptor pathway [6] (for review see [7]).

The p53-deficient HL-60 cell line demonstrated Hdm2 decrease as well as a putative Flt3 increase in response to IR or DNR (Fig. 5). The *FLT3 *gene in HL-60 is wild type [24], confirmed by sequencing of the juxtamembrane region and the kinase activation domain. Interestingly, lack of full length Flt3 protein in HL-60 has previously been reported [26], and we were not able to detect full length Flt3 in different batches of HL-60 cells from ATCC and DMZS (Fig. 3b). The protein bands between 50 and 100 kDa may be protein products from alternative splicing of *FLT3 *mRNA, as reported for the closely related platelet-derived growth factor alpha-receptor and *KIT *[35,36]. Additional work is clearly needed to address the possibility of alternative splicing of *FLT3 *in HL-60 and in AML cells in general.

Flt3-ITD is the strongest predictor for relapse of AML in therapy with anthracyclines [12], and is recently associated with enhanced DNA repair [13]. We demonstrated that the anti-apoptotic protein Bcl-2 was induced in MV4-11, HL-60 cells and primary AML cells during DNA damage therapy (Fig. 5). This could indicate that anthracyclines elicit an anti-apoptotic signal through Flt3. The anti-apoptotic signal may be particular strong in AML cells with a Flt3-ITD mutation including an Y591 duplication [14].

## Conclusion

In this study we show a concerted protein modulation of Flt3, Hdm2 and Mcl-1 after DNA damaging therapy in AML. IR resulted in decreased levels of Hdm2 and elevated levels of Flt3 and may involve p53 independent activities of Hdm2 acting on Flt3 as proposed for other receptor tyrosine kinases. The apoptotic response may depend on a persisting down regulation of Hdm2 and Mcl-1 [37]. Targeting of Flt3, Bcl-2/Bcl-X_L _and Mcl-1 is proposed to enhance the response of chemotherapy. Preclinical studies and early clinical trials that follow these principles are underway [38,39], and we believe that relevant biomarker examinations [3] including the proteins presented in this study may help to pinpoint the patients that will benefit from this enhanced therapy.

## Methods

### Cell culture

All patient studies were approved by the local ethical committee (REK Vest) and the Data Inspectorate, Norway. REK Vest is affiliated with the University of Bergen and Haukeland University Hospital. Samples were collected after informed consent. Patient data is overviewed in Table 1.

**Table 1:** Clinical and biological characteristics of AML patients

**Patients**	**Age**	**Sex**	**Previous malignant disease**	**FAB**	**Membrane molecules**					**Karyotype**	***FLT3 LM***	***FLT3 Asp835***	**Survival (Weeks)**
									
					**CD 13**	**CD 14**	**CD 15**	**CD 33**	**CD 34**				
# 1	72	M	Residive	AML M1	+	-	+	+	-	Normal	wt	wt	6
# 2	34	F	-	AML M5a	-	-	+	+	-	46 XX, t(9;11), (q22;q23)	wt	0.31	>24 (Tx)
# 3	55	M	-	Atypical	+	-	+	+	-	Multiple	wt	wt	>23

The patients were randomly selected; their clinical and biological data are included as background information.

Leukemic peripheral blood mononuclear cells (PBMC) were isolated by density gradient separation (Ficoll-Hypaque; Nycomed, Oslo, Norway) and were stored frozen in liquid nitrogen [40]. The percentage of blasts among leukemic PBMC exceeded 95% for all patients as judged by light microscopy of May-Grünwald-Giemsa stained cytospin smears [41]. PBMC were cultured in serum free conditions in StemSpan (Stem Cell Technologies, Vancouver, BC, Canada) at an average concentration of 2 × 10^6 ^cells per ml. Cells collected from patients during therapy followed the procedures as described by Anensen et al. 2006 [3].

The AML cell line NB4, kindly provided by Dr. Michel Lanotte (INSERM U-301, Hôpital St. Louis, Centre Hayem, Paris, France) [42], was cultured in RPMI 1640 (Sigma-Aldrich, Inc. St. Louis, MO, USA) with 10% fetal bovine serum (Foetal Calf Serum Gold, PAA Laboratories GmbH, Pasching, Austria) and penicillin/streptomycin 50 IU/50 μg per ml. Sequence analysis of both DNA strands of the NB4 cells used in this study confirmed wild type juxtamembrane region and activation loop of *FLT3*, and FISH analysis confirmed the presence of t(15;17) translocation. The same culture conditions as for NB4 were used for HL-60, purchased from DSMZ (Deutsche Sammlung von Mikroorganismen und Zellkulturen, Braunschweig, Germany). Reverse transcriptase PCR of HL-60 confirmed presence of normal length of *FLT3 *mRNA in the juxtamembranous region. The MV4-11 cell line was purchased from ATCC (American Type Culture Collection, Manassas, VA, USA) and cultured in IMDM (BioWhittaker, Cambrex Bio Science, Verviers, Belgium) with 10% FBS and penicillin/streptomycin 50 IU/50 μg per ml. The *FLT3 *gene in MV4-11 comprised a length mutation in the juxtamembrane region, and the t(4;11)(q21;q23) translocation was confirmed by FISH. The *TP53 *gene in MV4-11 is wild type according to data published [22] and the IARC *TP53 *Database [43]. The protein level of Flt3 in NB4 was approximately 50% of the level in MV4-11, estimated by Western blot and flow cytometry.

### Irradiation and chemotherapy treatment of cells

For irradiation induced DNA double strand breaks, samples were exposed to 25 Gray (Gy) from a Ce^137 ^source [11] and maintained in culture until samples were collected for Western blot analysis at time indicated. To secure that the observed effect was from the irradiation, the control samples were handled the same way as the exposed samples except for the actual irradiation. Collection of cells from AML patients under therapy and *in vitro *treatment of cells with daunorubicin was performed as previously described [3].

### Apoptosis assays

Cells were fixed in 2% paraformaldehyde solution containing the DNA specific nuclear stain Hoechst (Hoechst 33342, Invitrogen, Carlsbad, CA, USA; 10 μg/ml) and examined as previously described [33]. The number of normal and apoptotic nuclei was counted in an inverse fluorescence microscope (×400 magnification; Leica IRB, Leica Microsystems GmbH, Wetzlar, Germany). The mean number of three experiments was calculated together with the standard error of mean (standard deviation/√number of experiments). Nuclear staining with Hoechst of the cells treated with daunorubicin was not possible due to the strong fluorescence from the drug. These cells were fixed in 4% paraformaldehyde solution and their forward scatter and side scatter properties were examined by flow cytometry and used to determine the number of living cells. Flow cytometry was performed on a FacsCalibur flow cytometer (BD Biosciences, San Jose, CA, USA) and data analyses were carried out using the FlowJo software (Tree Star, Inc., Ashland, OR, USA).

### Western blotting

Samples for Western blotting were prepared by pelleting the cells (3–10 millions) and washing them twice in 0.9% NaCl following lysis in the following buffer: 10 mM Tris (pH 7.5), 1 mM EDTA, 400 mM NaCl, 10% glycerol, 0.5% NP40, 5 mM NaF, 0.5 mM sodium orthovanadate, 1 mM DTT, and 0.1 mM PMSF (50–200 μl lysis buffer per sample) and transfered to 1.5 ml tubes. The samples were homogenized by 20 strokes of a plastic mini homogenizer before centrifugation at 14000 × g for 20 minutes. Protein concentrations were determined using the Bradford protein assay, following the manufacturers instructions (Bio-Rad Laboratories, Inc., Hercules, CA, USA). The protein samples were added SDS loading buffer (Final: 1% SDS, 10% Glycerol, 12 mM Tris-HCl pH 6.8, 50 mM DTT and 0.1% Bromophenol Blue) and boiled for 10 minutes.

SDS-polyacrylamide gels, 10 or 12.5 % were loaded with 50–70 μg protein per well. After electrophoresis (100–200 V, 1–3 hours) and electroblotting (200 mA, o/n 4°C) the PVDF-membranes (HybondP, Amersham Biosciences, Oslo, Norway) were blocked for 1 hour in I-Block Blocking agent (Applied Biosystems, Foster City, CA, USA). Primary antibodies were incubated for 1–2 hours in room temperature or over night at 4°C followed by 1 hour washing in TBS-Tween. The antibodies Flt3 S-18, Hdm2 SMP-14, p53 BP53-12, Mcl-1 22, Bcl-2 ΔC 21 and Bax 2D2 were from Santa Cruz Biotechnology, CA, the Actin antibody AC-15 was from Abcam plc, Cambridge, UK and the Hdm2 antibodies 2A10 and IF2 were from Calbiochem, San Diego, CA, USA.

Secondary antibodies conjugated to horse radish peroxidase (Jackson ImmunoResearch laboratories, West Grove, PA, USA) were diluted in 4% fat-free dry milk in TBS-Tween and incubated 1 hour at room temperature. After washing for 1 hour with TBS-Tween, the membranes were developed using Supersignal^® ^West Pico or West Femto Chemiluminiscence Substate from Pierce Biotechnology Inc, Rockford, IL, USA according to the manufacturers' instructions. The membranes were imaged using a Kodak Image Station 2000R (Eastman Kodak Co., Lake Avenue, Rochester, NY, USA), and bands were quantified using the Kodak analysis software. Data were exported to Excel spreadsheet, corrected for background and loading control intensities and a Student's two-tailed *t *test was used for determination of significance.

### Real time PCR

Immediately after *in vitro *experiments, 5 × 10^6 ^cells were dissolved in RNAlater (Ambion Inc.) to stabilize and protect RNA and then stored at -80°C. RNAeasy plus mini kit (Qiagen Inc.) was used for isolation of total RNA. Cells were thawed, centrifuged and resuspended in RTL buffer and further procedures were followed according to manufacturer's instructions. RNA quality was tested on a 2100 Bioanalyzer (Agilent Technologies) and total RNA was quantified with a spectrophotometer for small aliquots (NanoDrop Technologies, Wilmington, DE, USA). cDNA were synthesized using the High-Capasity cDNA Archive Kit (Applied Biosystems, Foster City, CA) running 625 ng RNA in 50 μl total reaction volume. Real Time PCR was performed using assays-on-demand containing primers and FAM dye-labelled probes. Human GAPDH and β-Actin were used as endogenous controls. For Flt3 and Hdm2, assays Hs00174690_m1 and Hs00234753_m1 (Applied Biosystems) were used. TaqMan Universal PCR Master Mix (Applied Biosystems) was run with 2 μl cDNA in 10 μl total reaction volumes. The PCR was performed in a 384-well clear optical reaction plate on a 7900HT real time PCR system (Applied Biosystems). The calibrator sample in each experiment was used for standard curve dilution. All samples were run in three replicates and data were analyzed using the relative standard curve method as described by the manufacturer (Applied Biosystems).

## Competing interests

The author(s) declare that they have no competing interests.

## Authors' contributions

LW participated in the study design, performed experiments leading to Figure 1, 3 and 4 and wrote the manuscript. GS carried out the real-time PCR. IH contributed with the experiments in Figure 2. RH performed the sequencing of Flt3 and cytogenetics in patient cells and cell lines. ØB collected the patient material and participated in the design of the study. BTG conceived the study, participated in the study design and wrote the manuscript. All authors participated in the finalization of the manuscript, and read and approved the final manuscript for submission.
